# Metaplastic epithelial cells: origination from stem cells and promotion of intestinal inflammation

**DOI:** 10.1038/s41392-025-02165-3

**Published:** 2025-03-05

**Authors:** Tim Holland, Jochen Mattner

**Affiliations:** 1 Mikrobiologisches Institut - Klinische Mikrobiologie, Immunologie und Hygiene, Universitätsklinikum Erlangen and Friedrich-Alexander-Universität (FAU) Erlangen-Nürnberg, Erlangen, Germany; 2 FAU Profilzentrum Immunmedizin (FAU I-MED), FAU Erlangen-Nürnberg, Erlangen, Germany

**Keywords:** Inflammation, Gastrointestinal cancer

In a recent paper published in *Nature*, Oliver and colleagues report that pyloric gland metaplastic cells within the intestinal epithelium arise from crypt-based stem cells in response to chronic inflammatory irritation.^1^ These metaplastic cells to which the authors refer to as INFLAREs exhibit unique transcriptional signatures and promote inflammation-mediated tissue remodeling.

The human gastrointestinal tract consists of multiple specialized cell populations and organ systems that simultaneously control nutrient uptake and barrier immune homeostasis. Intestinal stem cells with long-term self-renewal and multilineage differentiation capacity maintain the intestinal epithelium and preserve tissue-specific cellular characteristics. However, one mature and differentiated cell type can replace another mature and differentiated cell type at tissue sites at which this cell type is usually not found, a process called metaplasia. Although Barret’s oesophagus and pyloric metaplasia represent histopathologically well-characterized examples for metaplastic tissue remodeling within the gastrointestinal tract, the origin and function of metaplastic cell transformation in inflammatory tissue damage have remained unknown.

To uncover this mystery, Oliver and colleagues established a gastrointestinal tract atlas into which they integrated single-cell RNA sequencing (scRNA-seq) data from their study and published results, containing a total of 25 datasets and 1.6 million cells derived from 271 donors.^1^ Therefore, they developed a novel method to automatically control the processed scRNA-seq datasets from various organ systems of the healthy human gastrointestinal tract and projected data from patients with six different gastrointestinal disorders onto the healthy reference atlas. These patient data included 2300 cells from eight patients with celiac disease, more than 280,000 cells from 49 patients with gastric or colorectal cancer (CRC) and more than 650,000 cells from 819 patients with inflammatory bowel disease (IBD).

Utilizing differential gene expression and individual, harmonized non-negative matrix factorization analyses, the authors characterized metaplastic Paneth cells in colonic and adjacent tissues of IBD patients in reference to the expression of distinct genetic patterns. As compared to indigenous Paneth cells in the irritated small intestine, metaplastic Paneth cells in affected colon tissues expressed more copies of genes that contribute to colonic tissue homeostasis and the control of bacterial growth. Thus, metaplastic Paneth cells in the large intestine express distinct genetic signatures that distinguish them from their native counterparts in the small intestine. However, metaplastic Paneth cells are not unique to IBD patients and can be also found in other diseases such as diverticulitis or radiation colitis.

**Fig. 1:**
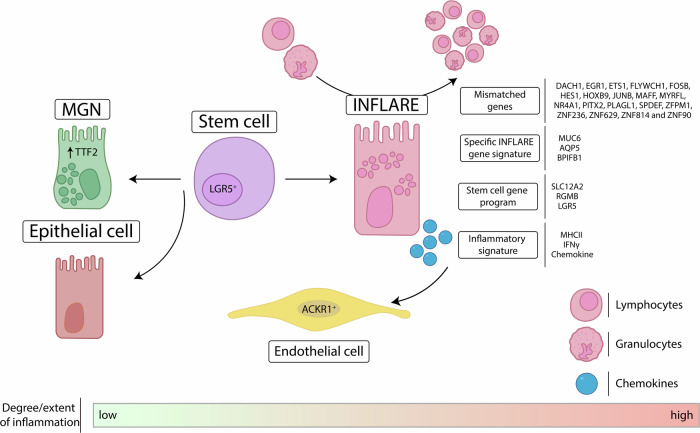
INFLAREs represent a metaplastic cell population with unique transcriptional signatures. INFLAREs originate from intestinal stem cells in response to inflammation-mediated irritation of the epithelial barrier. Although sharing some genetic similarities with native MGNs, epithelial cells and stem cells, INFLAREs distinguish themselves from all three subsets by a unique genetic signature that includes mismatched as well as specific INFLARE genes, a stem cell program and an inflammatory signature. Enhanced levels of chemokines, cytokine-induced inflammatory pathways and antigen-presenting molecules promote the recruitment of T cells and granulocytes suggesting INFLAREs as potential drivers of chronic inflammation and even malignant cell transformation. *ACKR1* atypical chemokine receptor 1, *INFLARE* inflammatory epithelial cell, *MGN* mucus gland neck cell

As novel metaplastic cell populations, Oliver and colleagues identified for the first time *MUC6*-positive mucous gland neck (MGN) cells and *MUC5AC*-expressing surface foveolar cells. Both cell subsets were enriched across tissues obtained from patients with Crohn’s disease (CD). In line with the tissue-specific restriction of inflammation which is characteristic for certain diseases, patients with ulcerative colitis exhibited an enrichment of both cell entities only in the large intestines. *MUC6*-expressing MGN cells and *MUC5AC*-positive surface foveolar cells shared transcriptional similarities to MGN cells from healthy tissues and phenotypically resembled cells of the Brunner’s glands that are usually found in the duodenum only proximal to the sphincter of Oddi.^2^ In contrast, *MUC6*-expressing MGN cells accumulated also distal the sphincter of Oddi and in higher numbers in patients with celiac disease before the initiation of treatment as well as in CRC-affected tissues. Based on the power of merging data to catalog infrequent cell populations, the authors described these *MUC6*-expressing cells as inflammatory epithelial cells (INFLAREs) that can be detected across all intestinal tissues, presumably in response to epithelial barrier irritation by chronic inflammation. Next to *MUC6*, they identified *PGC*, *AQP5*, and *BPIFB1* as INFLARE marker genes (Fig. 1). However, it remained unclear how frequent INFLAREs are in the intestines across individual patient cohorts.

Similar to other secretory and non-secretory cell populations of the gut, INFLAREs originated from LGR5-positive stem cells. Using gene-level pseudotime trajectory alignment of stem cells, Oliver and colleagues identified 19 transcription factors that were mismatched in INFLAREs (Fig. 1). Among them were cAMP response element-binding proteins that supposedly contribute to epithelial injury responses and metaplastic cell transformation in the stomach and pancreas.^3^ Although epithelial cells and INFLAREs shared some transcriptional programs, surface foveolar-like and goblet signatures as well as the expression of stemness genes distinguished INFLAREs from other mucous-secreting cells. Furthermore, cell-to-cell communication analyses suggested that the differential regulation of stem cell factors by INFLAREs might even contribute to metaplastic cell transformation (Fig. 1). Thus, INFLAREs are a plastic and peculiar cell lineage with exclusive transcriptional patterns and stem cell-like properties that the intestinal stem cell niche can generate in response to inflammation-mediated changes.

INFLAREs expressed antibacterial proteins and transcription factors involved in mucosal healing, in line with the assumption that metaplasia of epithelial cells might reflect an adaptive process to intestinal injury. Compared to MGN cells obtained from the duodenum or the stomach of healthy volunteers, however, INFLAREs also had genetic signatures that perpetuate chronic intestinal inflammation (Fig. 1). These inflammatory signatures included genes of cytokine-induced inflammatory programs, as well as MHC class II-related and IFN-γ-mediated pathways, and several chemokines that are critical for the recruitment of T lymphocytes, neutrophils and myeloid cells, similarly as observed in stem cells obtained from the ileum of CD patients. Moreover, Oliver and colleagues identified a mutual bond between *Ackr1*-expressing endothelial cells and INFLAREs in intestinal tissues obtained from CD patients. *Ackr1* encodes the atypical chemokine receptor 1, also known as Duffy antigen, which regulates the availability and distribution of chemokines due to sequestration, degeneration, or transcytosis.^4^ Since the expression of *Ackr1* by endothelial cells facilitates the transcytosis of monocytes into inflamed tissues and presumably mediates resistance to anti-TNF therapy in IBD patients,^5^ the close interaction of both, Ackr1-expressing vessels and INFLAREs might enhance inflammatory processes in intestinal diseases. In accordance with the enhanced expression of chemokine-, IFN-γ- and MHC class II-related signatures, different T cell subsets were detected in close proximity to INFLAREs within tissues of CD patients and patients with celiac disease.

In summary, Oliver and colleagues show that metaplastic cells within the intestinal epithelium originate from stem cells, exhibit transcriptional similarities to pyloric gland and Brunner’s gland cells, and promote immune cell recruitment, inflammation, and tissue remodeling.^1^ They refer to MUC6-expressing metaplastic cells from inflamed intestines as INFLAREs and introduce changes in epithelial stem cells as drivers of metaplastic lineage transformation. Consequently, INFLAREs trigger and sustain chronic inflammation and might even contribute to potential progression to IBD-associated neoplasia.

Future studies need to delineate the mechanisms and pathways underlying the metaplastic transformation of epithelial cells and the functional impact of stem cells in these processes. This will help to understand why only some patients develop metaplastic lesions and may guide the development of novel therapeutic approaches.
